# Implementation of a maternity hospital rotational thromboelastometry (ROTEM ^®^) guided transfusion strategy: a quality improvement study*

**DOI:** 10.1002/anr3.70028

**Published:** 2025-09-19

**Authors:** H. Tawfik, L. Pentony, R. M. O'Donovan, S. R. Mir, M. Tahir, T. Drew

**Affiliations:** ^1^ Department of Anaesthesia Rotunda Hospital Dublin Ireland; ^2^ Department of Laboratory Medicine Rotunda Hospital Dublin Ireland; ^3^ Department of Anaesthesia Rotunda and Beaumont Hospitals Dublin Ireland; ^4^ School of Medicine and Health Sciences Royal College of Surgeons in Ireland Dublin Ireland

**Keywords:** blood coagulation tests, fibrinogen, postpartum haemorrhage, ROTEM^®^ testing, viscoelastic testing

## Abstract

A rotational thromboelastometry (ROTEM^®^) guided transfusion strategy for obstetrics was implemented at our institution in September 2022. The aim of the strategy was to facilitate timely, targeted administration of coagulation products based on viscoelastic haemostatic testing, thereby reducing unnecessary transfusions. To improve compliance with the ROTEM^®^ strategy, an electronic decision tool was developed and integrated into a smartphone application, supported by departmental education and training. We subsequently analysed data on 944 women who experienced postpartum haemorrhage ≥ 1500 ml over a 5‐year period, comparing 1 year of data after the formal introduction of the ROTEM^®^‐guided transfusion strategy (post‐ROTEM^®^) with the previous 4 years, when a conventional transfusion strategy was in place based on standard laboratory tests (pre‐ROTEM^®^). Following implementation, the annual use of fibrinogen concentrate, Octaplas^®^ (Octapharma Pharmazeutika GmbH, Vienna, Austria) and platelets to treat PPH ≥ 1500 ml decreased by 46%, 72% and 79%, respectively, yielding a cost saving of €51,738. Compliance with evidence‐based fibrinogen transfusion triggers improved significantly (84% vs. 23%) and empirical product use was eliminated. There was no statistically significant difference in the proportion of women who progressed to severe haemorrhage (≥ 2000 ml) between groups: 97/238 (40%) in the post‐ROTEM^®^ group vs. 312/706 (44%) in the pre‐ROTEM group (p = 0.35). This quality improvement initiative demonstrated that embedding a ROTEM^®^‐guided transfusion strategy into clinical workflows, supported by a decision tool and staff training, can enhance adherence to evidence‐based practice, reduce unnecessary coagulation product use and generate substantial cost savings, without adversely affecting clinical outcomes. These findings may inform institutions seeking to optimise transfusion strategies in obstetric haemorrhage through structured implementation approaches.

## Introduction

The application of viscoelastic testing for detecting coagulation deficits and guiding transfusion during obstetric haemorrhage is increasing. However, high‐quality evidence supporting its superiority over conventional transfusion strategies remains limited [1]. National guidelines, including those from the National Institute for Health and Care Excellence (NICE), do not currently recommend the routine use of viscoelastic haemostatic assays (VHA) in this context [2]. Furthermore, major international bodies, such as the British Society for Haematology and the Royal College of Obstetricians and Gynaecologists, have also offered limited endorsement [3, 4]. Among available viscoelastic testing devices, rotational thromboelastometry (ROTEM^®^) is the most extensively studied in obstetric haemorrhage.

Given that coagulopathy is rare, affecting fewer than 5% of postpartum haemorrhage (PPH) cases, rapid identification of women who may require coagulation products is advantageous [5]. A key feature of ROTEM^®^ is its ability to quickly identify hypofibrinogenaemia via the FIBTEM A5 measurement, which reflects the clot amplitude 5 min after the clotting time. Fibrinogen is the first coagulation factor to decrease during PPH and both low Clauss fibrinogen levels (< 2 g.l^−1^) and FIBTEM A5 (< 12 mm) strongly predict the progression to severe PPH and the need for additional interventions [6, 7]. Addressing fibrinogen levels when they are low may improve clinical outcomes [8]. However, there is currently no evidence supporting the empirical administration of fibrinogen concentrate to women experiencing PPH [9, 10].

In September 2022, our institution implemented a ROTEM^®^‐guided transfusion strategy. To enhance adherence to this strategy, an electronic decision tool incorporating conditional logic was developed and integrated into a smartphone application, accessible here: https://form.jotform.com/230693021348048. Our group aimed to perform a pre‐ and post‐implementation analysis of the ROTEM^®^‐guided transfusion strategy, focusing on the total use of coagulation products for women experiencing PPH ≥ 1500 ml. We believe that our findings could assist clinicians considering the adoption of a similar programme at their institutions.

## Methods

A ROTEM^®^ Sigma device (Werfen, Barcelona, Spain) was evaluated on a trial basis in June 2022 at the Rotunda Hospital, Dublin, a tertiary maternity hospital with 8500 births annually, and purchased in August 2022. In September 2022, we introduced a formal ROTEM^®^‐guided transfusion protocol for PPH, supported by an electronic decision‐making tool employing conditional logic, integrated into an existing departmental smartphone application.

JotForm.com (San Francisco, US) was chosen due to its user‐friendly interface, robust conditional logic system and ability to create dynamic, interactive decision trees. The OBSCymru ROTEM thresholds for fibrinogen replacement were incorporated into a ROTEM^®^ algorithm tailored for local use, which was subsequently developed into a structured flowchart outlining key decision points based on ROTEM^®^ parameters [11, 12]. Each decision node was implemented as a conditional logic rule, ensuring that responses dynamically guided users through the appropriate decision pathways. The EXTEM CT threshold for Octaplas^®^ (Octapharma Pharmazeutika GmbH, Vienna, Austria) administration was set at 75 s in the presence of a normal fibrinogen level and 130 s in cases of hypofibrinogenemia. Based on the pathway followed, the ROTEM^®^ decision tool provided tailored management recommendations. The algorithm was tested using simulated ROTEM^®^ results to ensure correct pathway navigation and recommendation accuracy. Preliminary feedback indicated improved workflow efficiency and reduced cognitive load for clinicians. The ROTEM^®^‐guided transfusion algorithm and the electronic decision tool were approved by the department of anaesthesia, as well as the point‐of‐care and transfusion committees. ROTEM^®^ was added to the hospitals PPH management algorithm. Regular audits were established to ensure ongoing adherence and governance compliance. As the ROTEM^®^ clinical decision tool was designed to support, but not replace, internal clinical decision‐making, it was not classified as a medical device and therefore did not require regulatory approval.

All anaesthetists in our department received comprehensive training in the use of ROTEM^®^ and the associated electronic decision support tool. The training programme consisted of structured user guidelines, a didactic lecture and practical hands‐on sessions. A 30‐min lecture was delivered by a consultant anaesthetist in August 2022. This session provided an overview of the evidence underpinning ROTEM^®^‐guided transfusion strategies, including thresholds for intervention and explored the OBSCymru guideline in detail. Key decision‐making principles were emphasised, particularly the importance of applying ROTEM^®^ in a context‐specific manner. Clinicians were advised to consider factors affecting maternal physiology and the nature of the haemorrhage, such as maternal weight, estimated blood volume and the underlying cause of PPH [13, 14]. Although a blood loss of 1500 ml was broadly recognised as a threshold for increased concern and ROTEM^®^ measurement, emphasis was placed on using clinical judgement to guide the timing of ROTEM^®^ rather than relying solely on fixed volume criteria. This pragmatic approach was designed to reflect real‐world decision‐making in obstetric emergencies and was consistent with our institutional policy. Practical training included two 25‐min sessions demonstrating the ROTEM^®^ machine and the electronic decision tool, supported by worked clinical examples. A multidisciplinary simulation session followed which featured a PPH scenario, integrating ROTEM^®^ data interpretation and use of the decision tool in real time.

Training on the use of ROTEM^®^ was also embedded within the institutional bi‐monthly multidisciplinary obstetric emergencies course, modelled on the PRactical Obstetric Multi‐Professional Training (PROMPT) framework. ROTEM^®^ was incorporated into both the course manual and simulation scenarios to ensure its integration into routine obstetric emergency management. Residents were provided with materials accessible via their smartphones, including the clinical decision tool and an instructional video on performing the ROTEM^®^ test. All user guidelines and resources were made available electronically through the departmental smartphone application, ensuring continuous accessibility. Refresher training was provided every 6 months, with additional sessions available on request to support continuous professional development.

In 2023, a retrospective analysis of institutional blood product usage for the management of PPH was approved by the Research Ethics and Audit Committee of the Rotunda Hospital (REC 23‐042). We analysed data on 944 women who experienced PPH ≥ 1500 ml over a 5‐year period. We compared 1 year of data after ROTEM^®^ strategy implementation (post‐ROTEM^®^) with the previous 4 years using a conventional transfusion strategy (pre‐ROTEM^®^). The primary outcome was overall consumption of blood coagulation products. Secondary outcomes included compliance with transfusion triggers (Clauss fibrinogen < 2 g.l^−1^, FIBTEM A5 < 12 mm), as well as progression of haemorrhage from ≥ 1500 ml to ≥ 2000 ml. Correlation analyses were also performed between FIBTEM A5, laboratory Clauss fibrinogen and measured blood loss (MBL).

Data were analysed and figures were produced using Prism (version 7; GraphPad, La Jolla, CA, USA). Continuous data are presented as mean (SD) where appropriate. Proportions were compared using the Chi‐squared test. A Pearson's correlation analysis was used to determine the correlation between Clauss fibrinogen and FIBTEM A5 with MBL.

## Results

The incidence of PPH, defined as MBL ≥ 1500 ml, was higher in the post‐ROTEM^®^ group compared to the conventional group: 238/8087 (2.9%) vs. 706/33,612 (2.1%), p < 0.001. Utilisation of ROTEM^®^ increased with haemorrhage severity: 155 of 238 women (65%) with MBL ≥ 1500 ml underwent ROTEM^®^ assessment and all 97 (100%) with MBL ≥ 2000 ml received ROTEM^®^‐guided evaluation. Additionally, 28/484 (6%) of women with MBL between 1000 and 1500 ml underwent ROTEM^®^ testing. Among all women who received a ROTEM^®^ test, the mean MBL was 1997 ml (SD 1429 ml), indicating substantial variability in blood loss within this group. Of the ROTEM^®^ tests performed, 45 of 183 (25%) showed a FIBTEM A5 value < 12 mm.

Post‐ROTEM^®^‐guided care was associated with a marked reduction in blood product transfusion. The mean annual use of fibrinogen concentrate during PPH ≥ 1500 ml fell from 175 g (€93,170) in the pre‐ROTEM^®^ group to 94 g (€50,045) in the post‐ROTEM^®^ group, representing a 46% reduction. Similarly, Octaplas^®^ (Octapharma Pharmazeutika GmbH, Vienna, Austria) use declined from 44 units (€5046) to 12 units (€1392), representing a 72% decrease; and pooled platelet use dropped from 9.75 units (€6240) to 2 units (€1280), representing a 79% reduction. Among the patients who experienced PPH ≥ 1500 ml, fibrinogen concentrate was administered to 31/228 (13%) in the post‐ROTEM^®^ group compared to 152/706 (22%) in the pre‐ROTEM^®^ group (p = 0.004). Octaplas^®^ was given to 4/238 (1.7%) women in the post‐ROTEM^®^ group compared with 41/706 (5.8%) in the pre‐ROTEM^®^ group (p = 0.01) and platelets were administered to 2/238 (0.8%) women in the post‐ROTEM^®^ group vs. 14/706 (2%) in the pre‐ROTEM^®^ group (p = 0.23).

Among women who received fibrinogen over the study period, the proportion meeting evidence‐based transfusion criteria, defined as Clauss fibrinogen < 2 g.l^−1^ or FIBTEM A5 < 12 mm, increased from 23% in the pre‐ROTEM^®^ group to 84% in the post‐ROTEM^®^ group. No women in the post‐ROTEM^®^ group received empirical fibrinogen (transfusion without prior fibrinogen testing), compared with 23% in the pre‐ROTEM^®^ group. Moreover, inappropriate administration of fibrinogen, defined as transfusion despite test results above threshold (Clauss fibrinogen ≥ 2 g.l^−1^ or FIBTEM ≥ 12 mm), declined from 51% in the pre‐ROTEM^®^ group to 16% in the post‐ROTEM^®^ group. The results of our study are summarised in Table 1.

**Table 1:** Summary of results.

	Pre‐ROTEM^®^	Post‐ROTEM^®^	p‐Value
Births	33,612	8087	
PPH ≥ 1000 ml	2066 (6.1%)	723 (8.9%)	< 0.001*
PPH ≥ 1500 ml	706 (2.1%)	238 (2.9%)	< 0.001*
Had ROTEM^®^ guided treatment			
≥ 1000 ml	0	183/723 (25%)	
1000–1500 ml	0	28/484 (6%)	
≥ 1500 ml	0	155/238 (65%)	
≥ 2000 ml	0	97/97 (100%)	
Proportion of patients with PPH ≥ 1500 ml who received fibrinogen concentrate	152/706 (22%)	31/238 (13%)	0.004*
Proportion of patients with PPH ≥ 1500 ml who received Octaplas^®^	41/706 (5.8%)	4/238 (1.7%)	0.01*
Proportion of patients with PPH ≥ 1500 ml who received platelets	14/706 (2%)	2/238 (<1%)	0.23

PPH, postpartum haemorrhage.

* denotes statistical significance at p < 0.05.

**Figure 1:**
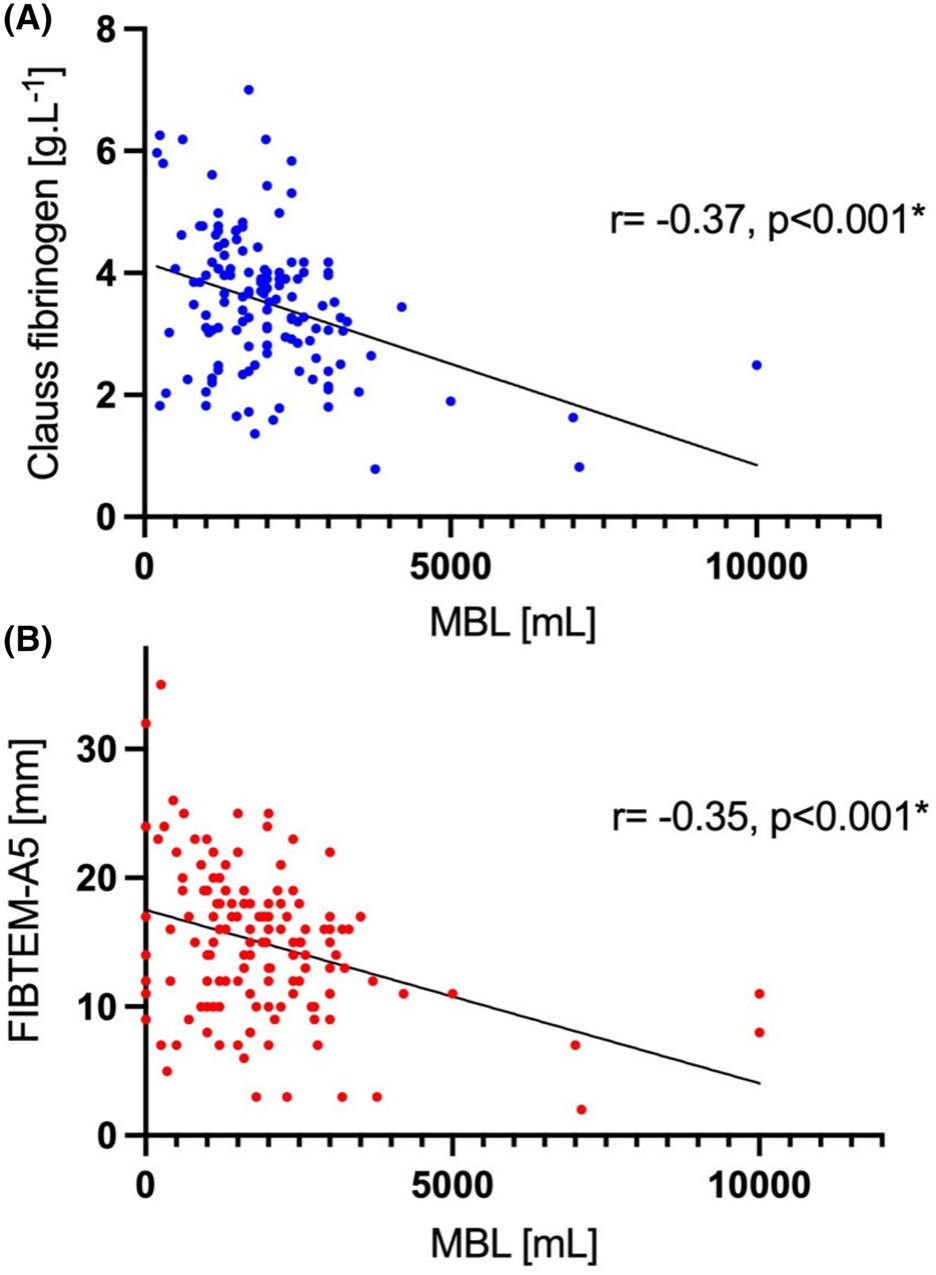
Correlation between (A) Clauss fibrinogen and (B) FIBTEM A5 with measured blood loss. MBL, measured blood loss. *denotes statistical significance at p < 0.05.

There was no significant difference between the groups in the proportion of women progressing from ≥ 1500 ml to ≥ 2000 ml blood loss (post‐ROTEM^®^ 97/238, 40% vs. pre‐ROTEM^®^ 312/706, 44%; p = 0.35). Finally, both FIBTEM A5 and Clauss fibrinogen values demonstrated a weak inverse correlation with MBL (FIBTEM A5 r = −0.35, p < 0.001; Clauss fibrinogen r = −0.37, p < 0.001) (Figure 1).

## Discussion

Our data show that the implementation of a standardised ROTEM^®^‐guided transfusion strategy coincided with a notable reduction in the use of blood coagulation products for PPH. Notably, this reduction occurred despite an increase in the incidence of PPH, which is a concerning national trend [15]. The ROTEM^®^‐guided strategy was associated with cost savings of approximately €52,000 over 1 year, likely as a result of increased adherence to evidence‐based transfusion triggers, rather than the empirical administration of coagulation products. These findings may be useful to clinicians considering a transition to ROTEM^®^‐guided transfusion strategies and seeking evidence to support the associated cost implications.

While the ROTEM^®^ machine served as the visible centrepiece of the transfusion strategy, our experience suggests that its successful implementation relied heavily on broader quality improvement principles. Key enablers included early and sustained engagement from clinical champions, visibility of the project across the institution and the accessibility of practical training materials. Embedding ROTEM^®^ interpretation into a smartphone app helped integrate the change into daily workflows, while simulation‐based education and regular refresher sessions reinforced practice change. The creation of a simple, accessible decision tool reduced cognitive load during emergencies, making it easier for clinicians, especially rotating doctors, to engage with the protocol. Notably, our results likely reflect the cumulative impact of these interventions, rather than the introduction of ROTEM^®^ alone. This underscores the importance of structured implementation strategies, ongoing education and leadership in translating evidence‐based tools into consistent clinical practice.

In most cases of PPH, coagulation factor levels remain within the normal range unless a consumptive process is present, such as placental abruption or amniotic fluid embolism. As a result, prolonged EXTEM CT due to factor depletion is uncommon in the early phase of PPH, whereas hypofibrinogenaemia occurs more frequently. Many coagulopathies which appear to involve factor deficiency can often be corrected by restoring fibrin polymerisation alone. There is currently no strong evidence about when to transfuse plasma‐based products based on ROTEM results [16]. Although current guidance recommends administration of FFP or Octaplas^®^ after fibrinogen replacement, we adopted a more conservative strategy informed by prior experience with early factor deficiency in major PPH and amniotic fluid embolism. Similar to practices in other centres, we implemented an EXTEM CT threshold for FFP administration which varied according to fibrinogen concentration: if fibrinogen was within the normal range, Octaplas^®^ was administered when EXTEM CT > 75 s; in the presence of hypofibrinogenaemia, a higher threshold of > 130 s was applied.

Integrating ROTEM^®^ algorithms into clinical practice presents challenges, particularly due to the complexity of interpreting its output, which comprises more than 25 data points per test. This complexity is compounded by the high‐pressure context of managing critical events, often occurring out of hours. Recognising the safety risks associated with incorrect interpretation by rotating non‐consultant anaesthetists, as well as reluctance to use the device from more junior anaesthetic residents, our decision‐support tool was designed to aid the interpretation of ROTEM^®^ results. Use of ROTEM^®^ was strongly associated with the severity of postpartum bleeding, with universal uptake in cases of MBL ≥ 2000 ml and selective application in moderate haemorrhage. Compliance of 65% for the use of ROTEM for PPH ≥ 1500 ml is similar to that reported elsewhere [14]. Viscoelastic haemostatic assays in women with MBL < 1500 ml indicate that factors beyond quantitative blood loss, such as haemodynamic instability or clinical suspicion of coagulopathy, may additionally inform decision‐making. Our data suggest that increased clinical awareness and training, along with enhanced access to and interpretation of ROTEM^®^ (via the decision tool), improved adherence to evidence‐based transfusion triggers and consequently reduced unnecessary coagulation product use. As this was an observational study, our results cannot establish causality. Other contributing factors, such as increased general clinical awareness or education on haemorrhage management from outside our institution, may also have contributed to improved transfusion practices.

Two recently published randomised controlled trials have compared ROTEM^®^‐guided transfusion to transfusion based on conventional laboratory values. One study randomised 49 women with PPH following vaginal or caesarean birth to groups with either disclosed or blinded ROTEM^®^ results. It found comparable transfusion rates between the groups, although the study was underpowered due to early termination from slow accrual [17]. Another study randomised 60 women with PPH ≥ 1500 ml to ROTEM^®^‐guided or conventional treatment, with 54 women included in the final analysis. No differences were observed in the primary outcome of red blood cell units transfused. These findings suggest that larger trials are needed to demonstrate clinically significant improvements in patient outcomes [18].

There are several limitations to our quality improvement project results. We did not conduct a formal baseline vs. post‐implementation knowledge assessment of staff or anaesthetists' views on the ROTEM^®^, however informal feedback indicated improved understanding and compliance post‐training. All ROTEM^®^ use at our institution was electronically recorded, ensuring accurate capture. Feedback from doctors was that the use of the electronic decision tool was universal. However, it is conceivable that clinicians sometimes informally reviewed ROTEM^®^ results without fully completing the electronic decision tool pathway.

It is important to note that our institution used fibrinogen concentrate, which cost €532 per gram during the study period. The findings may not therefore be directly applicable to institutions which use cryoprecipitate. Additionally, Werfen, the manufacturer of ROTEM^®^, has recently issued a field safety notice regarding changes to the FIBTEM assay. A second reagent has been added, altering the relationship between FIBTEM A5 and Clauss fibrinogen, prompting some guidelines to recommend a revised fibrinogen transfusion threshold of ≤ 8 mm [19]. Our decision support tool has been modified since completion of this study to reflect these recommendations.

In conclusion, implementing an electronic‐based ROTEM^®^‐guided transfusion strategy coincided with a substantial reduction in the use of blood coagulation products, particularly fibrinogen concentrate, at our institution. We suggest these reductions were primarily driven by training and the rapid availability of ROTEM^®^ results, enabling more targeted and evidence‐based transfusion practices, as evidenced by a reduction in empiric coagulation product administration. We advocate for the increased use of electronic decision‐support tools to enhance the usability and accuracy of ROTEM^®^ interpretation.
